# *catena*-Poly[[[bis­(4-methyl­benzoato-κ^2^ *O*,*O*′)zinc(II)]-μ-4,4′-bipyridine-κ^2^ *N*:*N*′] tetra­hydrate]

**DOI:** 10.1107/S1600536809005571

**Published:** 2009-02-28

**Authors:** Xiao-Yan Li, Yin-Feng Han, Ji-Kun Li

**Affiliations:** a Department of Chemistry, Taishan University, 271000 Taishan, Shandong, People’s Republic of China; b Department of Materials Science and Chemical Engineering, Taishan University, 271021 Taian, Shandong, People’s Republic of China

## Abstract

The asymmetric unit of the title compound, {[Zn(C_7_H_7_O_2_)_2_(C_10_H_8_N_2_)]·4H_2_O}_*n*_, contains a highly distorted octa­hedral Zn^II^ metal center strongly coordinated by two N atoms of two 4,4′-bipyridine (4,4′-bipy) ligands and chelated by two 4-methyl­benzoate anions. The crystallographic inversion center and glide plane present at the center of the C—C single bond of 4,4′-bipy, along with the *cis* coordination motif of the 4,4′-bipy, lead to one-dimensional zigzag chains. There are a large number of water mol­ecules in the crystal structure, which also form one-dimensional chains through O—H⋯O hydrogen bonds.

## Related literature

For inorganic–organic hybrid frameworks containing *d*-block transition metal ions and pyridyl ligands, see: Batten & Robson (1998 ▶); Horikoshi & Mochida (2006 ▶); Fujita *et al.* (1994 ▶); Luan *et al.* (2005 ▶); Tao *et al.* (2002 ▶).
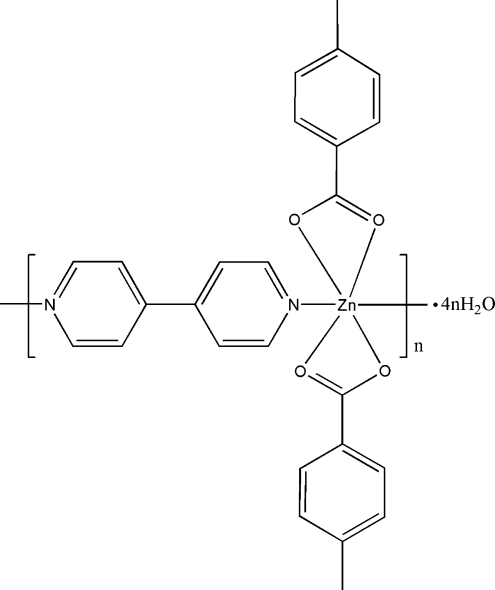


## Experimental

### 

#### Crystal data

[Zn(C_7_H_7_O_2_)_2_(C_10_H_8_N_2_)]·4H_2_O
*M* *_r_* = 563.89
Monoclinic, 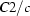

*a* = 12.024 (5) Å
*b* = 18.803 (8) Å
*c* = 12.283 (5) Å
β = 98.063 (6)°
*V* = 2750 (2) Å^3^
*Z* = 4
Mo *K*α radiation
μ = 0.94 mm^−1^
*T* = 298 K
0.25 × 0.23 × 0.22 mm

#### Data collection

Bruker SMART APEX area-detector diffractometer
Absorption correction: multi-scan (*SADABS*; Sheldrick, 2003 ▶) *T*_min_ = 0.799, *T*_max_ = 0.820
9512 measured reflections
2439 independent reflections
2306 reflections with *I* > 2σ(*I*)
*R*_int_ = 0.065

#### Refinement

*R*[*F* ^2^ > 2σ(*F* ^2^)] = 0.042
*wR*(*F* ^2^) = 0.119
*S* = 0.90
2439 reflections
169 parameters
H-atom parameters constrained
Δρ_max_ = 0.41 e Å^−3^
Δρ_min_ = −0.36 e Å^−3^

### 

Data collection: *SMART* (Bruker, 2003 ▶); cell refinement: *SAINT-Plus* (Bruker, 2003 ▶); data reduction: *SAINT-Plus*; program(s) used to solve structure: *SHELXS97* (Sheldrick, 2008 ▶); program(s) used to refine structure: *SHELXL97* (Sheldrick, 2008 ▶); molecular graphics: *SHELXTL* (Sheldrick, 2008 ▶); software used to prepare material for publication: *SHELXTL*.

## Supplementary Material

Crystal structure: contains datablocks global, I. DOI: 10.1107/S1600536809005571/ez2143sup1.cif

Structure factors: contains datablocks I. DOI: 10.1107/S1600536809005571/ez2143Isup2.hkl

Additional supplementary materials:  crystallographic information; 3D view; checkCIF report

## Figures and Tables

**Table 1:** Hydrogen-bond geometry (Å, °)

*D*—H⋯*A*	*D*—H	H⋯*A*	*D*⋯*A*	*D*—H⋯*A*
O4—H4*B*⋯O4^i^	0.85	1.93	2.761 (7)	167
O3—H3*A*⋯O2^ii^	0.85	1.93	2.777 (4)	179

Symmetry codes: (i) 
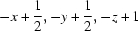
; (ii) 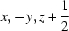.

**Fig. 1.**
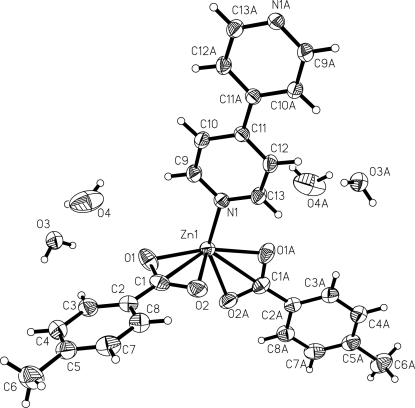
The structure of (I) showing 30% probability displacement ellipsoids and the atom-numbering scheme (A: -x, y, 0.5 - z).

**Fig. 2.**
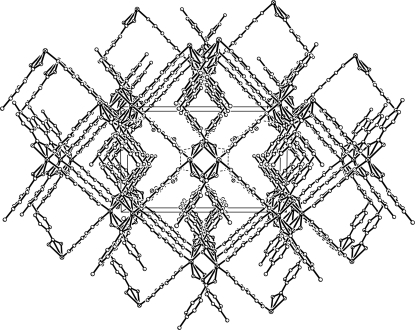
The zig-zag chains of (I), with dashed lines indicating O—H···O hydrogen bonds.

## supplementary crystallographic information

### Comment

Recently much attention has been paid to inorganic-organic hybrid frameworks that contain d-block transition metal ions and pyridyl ligands (Batten & Robson, 1998; Horikoshi & Mochida, 2006). These inorganic-organic hybrid frameworks form a wide range of interesting network topologies, such as chains, ladders or grids (Fujita *et al.*, 1994; Luan *et al.* 2005). In these compounds, the combination of 4,4'-bipy and carboxylate ligands is largely directed toward obtaining interesting topologies (Tao *et al.*, 2002). Here, we report the synthesis and crystal structure of the title complex, 1, which combines 4,4'-bipy and 4-methylbenzoate ligands.

Single-crystal X-ray diffraction studies reveal that the asymmetric unit contains the basic building block of 1, C_26_H_22_N_2_O_4_Cd.4(H_2_O), as shown in Figure 1. The highly distorted octahedral Zn^II^ metal center is strongly coordinated to two N atoms of two 4,4'-bipy ligands [Zn—N, 2.064 (2) Å] and chelated to two 4-methylbenzoate anions [Zn1—O1, 2.159 (3) Å and Zn1—O2, 2.261 (3) Å]. The crystallographic inversion center and glide plane present at the center of the carbon-carbon single bond of the 4,4'-bipy ligand generate one-dimensional zig-zag coordination polymers. The zig-zag chains run approximately in parallel, as shown in Figure 2. The N1—Zn1—N1A [A: -*x*, *y*, 0.5 - *z*] angle of 105.4 (3)°, contributes to the chelate formation of the 4-methylbenzoate anions. The dihedral angles between the planes through 4,4'-bipy and 4-methylbenzoate are 84.68 (2)°. The Zn···Zn distances separated by the 4,4'-bipy are 11.20 (2) Å. The large number of included water molecules form one-dimensional chains through O—H···O hydrogen bonds.

### Experimental

Zinc dichloride hexahydrate (2 mmol), 4-methylbenzoic acid (4 mmol) and 4,4'-bipy (2 mmol) were dissolved in a 3:1 ethanol-water solution (20 ml). Aqueous 0.1 *M* sodium hydroxide was added until the solution registered a pH of 7. The solution was set aside for the growth of crystals over several days. Anal. calc. for C_26_H_30_N_2_O_8_Zn: C 55.38, H 5.36, N 4.97%. Found: C 55.25, H 5.40, N 4.86%.

### Refinement

All H atoms bound to C were placed in idealized positions (C—H = 0.93—0.97 Å) and refined as riding atoms, with the *U*_iso_(H) = 1.2 or 1.5*U*_eq_(C). Some H atoms bound to O were treated for 50:50 disorder, with all O—H bond lengths of 0.850 and *U*_iso_(H) = 1.2 *U*_eq_(O).

### Crystal data

**Table:**

[Zn(C_7_H_7_O_2_)_2_(C_10_H_8_N_2_)]·4H_2_O	*F*(000) = 1176
*M**_r_* = 563.89	*D*_x_ = 1.362 Mg m^−^^3^
Monoclinic, *C*2/*c*	Mo *K*α radiation, λ = 0.71073 Å
Hall symbol: -C 2yc	Cell parameters from 921 reflections
*a* = 12.024 (5) Å	θ = 2.7–28.1°
*b* = 18.803 (8) Å	µ = 0.94 mm^−^^1^
*c* = 12.283 (5) Å	*T* = 298 K
β = 98.063 (6)°	Block, yellow
*V* = 2750 (2) Å^3^	0.25 × 0.23 × 0.22 mm
*Z* = 4	

### Data collection

**Table:**

Bruker APEX area-detector diffractometer	2439 independent reflections
Radiation source: fine-focus sealed tube	2306 reflections with *I* > 2σ(*I*)
graphite	*R*_int_ = 0.065
φ and ω scans	θ_max_ = 25.0°, θ_min_ = 2.0°
Absorption correction: multi-scan (*SADABS*; Sheldrick, 2003)	*h* = −14→14
*T*_min_ = 0.799, *T*_max_ = 0.820	*k* = −22→22
9512 measured reflections	*l* = −14→14

### Refinement

**Table:**

Refinement on *F*^2^	Primary atom site location: structure-invariant direct methods
Least-squares matrix: full	Secondary atom site location: difference Fourier map
*R*[*F*^2^ > 2σ(*F*^2^)] = 0.042	Hydrogen site location: inferred from neighbouring sites
*wR*(*F*^2^) = 0.119	H-atom parameters constrained
*S* = 0.90	*w* = 1/[σ^2^(*F*_o_^2^) + (0.0704*P*)^2^ + 5.1543*P*] where *P* = (*F*_o_^2^ + 2*F*_c_^2^)/3
2439 reflections	(Δ/σ)_max_ < 0.001
169 parameters	Δρ_max_ = 0.41 e Å^−^^3^
0 restraints	Δρ_min_ = −0.36 e Å^−^^3^

### Special details

**Table:**

Geometry. All e.s.d.'s (except the e.s.d. in the dihedral angle between two l.s. planes) are estimated using the full covariance matrix. The cell e.s.d.'s are taken into account individually in the estimation of e.s.d.'s in distances, angles and torsion angles; correlations between e.s.d.'s in cell parameters are only used when they are defined by crystal symmetry. An approximate (isotropic) treatment of cell e.s.d.'s is used for estimating e.s.d.'s involving l.s. planes.
Refinement. Refinement of *F*^2^ against ALL reflections. The weighted *R*-factor *wR* and goodness of fit *S* are based on *F*^2^, conventional *R*-factors *R* are based on *F*, with *F* set to zero for negative *F*^2^. The threshold expression of *F*^2^ > σ(*F*^2^) is used only for calculating *R*-factors(gt) *etc*. and is not relevant to the choice of reflections for refinement. *R*-factors based on *F*^2^ are statistically about twice as large as those based on *F*, and *R*- factors based on ALL data will be even larger.

### Fractional atomic coordinates and isotropic or equivalent isotropic displacement parameters (Å^2^)

**Table:**

	*x*	*y*	*z*	*U*_iso_*/*U*_eq_	Occ. (<1)
Zn1	0.0000	0.07933 (2)	0.2500	0.04633 (19)	
O1	0.1310 (2)	0.06512 (15)	0.3876 (2)	0.0757 (7)	
O2	0.1328 (2)	−0.00574 (12)	0.2496 (2)	0.0744 (6)	
N1	0.0873 (2)	0.14583 (12)	0.15842 (18)	0.0468 (5)	
C1	0.1739 (3)	0.01398 (17)	0.3444 (3)	0.0587 (8)	
C2	0.2720 (2)	−0.02359 (15)	0.4060 (3)	0.0529 (7)	
C3	0.2954 (3)	−0.01735 (18)	0.5189 (3)	0.0658 (8)	
H3	0.2522	0.0127	0.5560	0.079*	
C4	0.3823 (3)	−0.0552 (2)	0.5773 (3)	0.0734 (10)	
H4	0.3961	−0.0507	0.6534	0.088*	
C5	0.4488 (3)	−0.09957 (19)	0.5252 (3)	0.0695 (9)	
C6	0.5420 (4)	−0.1422 (3)	0.5892 (4)	0.1006 (15)	
H6A	0.5905	−0.1603	0.5399	0.151*	
H6B	0.5105	−0.1811	0.6252	0.151*	
H6C	0.5844	−0.1123	0.6432	0.151*	
C7	0.4265 (3)	−0.1047 (2)	0.4118 (4)	0.0744 (10)	
H7	0.4717	−0.1335	0.3748	0.089*	
C8	0.3386 (3)	−0.06778 (18)	0.3519 (3)	0.0636 (8)	
H8	0.3244	−0.0727	0.2759	0.076*	
C9	0.1610 (3)	0.19327 (18)	0.2051 (2)	0.0670 (9)	
H9	0.1695	0.1984	0.2811	0.080*	
C10	0.2254 (3)	0.23496 (18)	0.1467 (2)	0.0650 (9)	
H10	0.2751	0.2678	0.1831	0.078*	
C11	0.2161 (2)	0.22802 (13)	0.0331 (2)	0.0436 (6)	
C12	0.1406 (3)	0.17860 (16)	−0.0138 (2)	0.0528 (7)	
H12	0.1312	0.1717	−0.0896	0.063*	
C13	0.0785 (3)	0.13905 (17)	0.0500 (2)	0.0542 (7)	
H13	0.0280	0.1059	0.0155	0.065*	
O3	0.0969 (2)	0.14890 (13)	0.70161 (19)	0.0729 (7)	
H3A	0.1086	0.1050	0.7156	0.088*	
H3B	0.1308	0.1602	0.6479	0.088*	0.50
H3C	0.0269	0.1566	0.6855	0.088*	0.50
O4	0.2054 (4)	0.1867 (2)	0.5310 (4)	0.1517 (19)	
H4A	0.1876	0.1557	0.4813	0.182*	
H4B	0.2322	0.2228	0.5021	0.182*	0.50
H4C	0.1485	0.1983	0.5610	0.182*	0.50

### Atomic displacement parameters (Å^2^)

**Table:**

	*U*^11^	*U*^22^	*U*^33^	*U*^12^	*U*^13^	*U*^23^
Zn1	0.0510 (3)	0.0440 (3)	0.0457 (3)	0.000	0.01289 (19)	0.000
O1	0.0696 (15)	0.0948 (17)	0.0643 (12)	0.0361 (14)	0.0153 (9)	0.0181 (11)
O2	0.0714 (15)	0.0537 (12)	0.0922 (16)	0.0085 (9)	−0.0095 (13)	0.0086 (10)
N1	0.0511 (13)	0.0467 (12)	0.0435 (12)	−0.0026 (10)	0.0095 (10)	0.0015 (9)
C1	0.0548 (18)	0.0560 (17)	0.069 (2)	0.0016 (14)	0.0215 (15)	0.0166 (15)
C2	0.0486 (16)	0.0478 (15)	0.0642 (18)	0.0014 (12)	0.0150 (13)	0.0029 (13)
C3	0.075 (2)	0.0558 (17)	0.067 (2)	0.0092 (16)	0.0112 (16)	−0.0018 (15)
C4	0.082 (3)	0.0650 (19)	0.069 (2)	0.0048 (19)	−0.0048 (18)	0.0014 (17)
C5	0.0543 (19)	0.0577 (18)	0.093 (3)	0.0000 (15)	−0.0008 (18)	0.0053 (18)
C6	0.074 (3)	0.087 (3)	0.134 (4)	0.015 (2)	−0.008 (3)	0.015 (3)
C7	0.058 (2)	0.062 (2)	0.107 (3)	0.0102 (16)	0.0253 (19)	−0.005 (2)
C8	0.062 (2)	0.0624 (18)	0.069 (2)	0.0046 (15)	0.0179 (16)	−0.0025 (15)
C9	0.093 (3)	0.070 (2)	0.0378 (14)	−0.0261 (18)	0.0110 (15)	−0.0016 (14)
C10	0.086 (2)	0.0665 (19)	0.0415 (15)	−0.0329 (17)	0.0068 (14)	−0.0032 (13)
C11	0.0483 (14)	0.0419 (13)	0.0410 (13)	0.0010 (11)	0.0071 (11)	0.0014 (10)
C12	0.0562 (17)	0.0639 (17)	0.0387 (13)	−0.0126 (14)	0.0082 (12)	−0.0048 (12)
C13	0.0552 (17)	0.0625 (17)	0.0465 (15)	−0.0146 (14)	0.0122 (12)	−0.0072 (13)
O3	0.0812 (17)	0.0674 (14)	0.0682 (15)	0.0017 (12)	0.0035 (12)	0.0014 (11)
O4	0.194 (4)	0.105 (3)	0.183 (4)	−0.051 (3)	0.121 (4)	−0.034 (3)

### Geometric parameters (Å, °)

**Table:**

Zn1—N1^i^	2.064 (2)	C6—H6A	0.9600
Zn1—N1	2.064 (2)	C6—H6B	0.9600
Zn1—O1	2.159 (3)	C6—H6C	0.9600
Zn1—O1^i^	2.159 (3)	C7—C8	1.386 (5)
Zn1—O2	2.261 (3)	C7—H7	0.9300
Zn1—O2^i^	2.261 (3)	C8—H8	0.9300
Zn1—C1^i^	2.559 (3)	C9—C10	1.372 (4)
Zn1—C1	2.559 (3)	C9—H9	0.9300
O1—C1	1.245 (4)	C10—C11	1.390 (4)
O2—C1	1.255 (4)	C10—H10	0.9300
N1—C9	1.330 (4)	C11—C12	1.369 (4)
N1—C13	1.328 (4)	C11—C11^ii^	1.481 (5)
C1—C2	1.487 (4)	C12—C13	1.374 (4)
C2—C8	1.386 (4)	C12—H12	0.9300
C2—C3	1.381 (5)	C13—H13	0.9300
C3—C4	1.379 (5)	O3—H3A	0.8501
C3—H3	0.9300	O3—H3B	0.8500
C4—C5	1.374 (6)	O3—H3C	0.8500
C4—H4	0.9300	O4—H4A	0.8500
C5—C7	1.385 (6)	O4—H4B	0.8501
C5—C6	1.506 (5)	O4—H4C	0.8501
			
N1^i^—Zn1—N1	105.44 (13)	C4—C3—C2	120.7 (3)
N1^i^—Zn1—O1	91.10 (10)	C4—C3—H3	119.6
N1—Zn1—O1	97.52 (10)	C2—C3—H3	119.6
N1^i^—Zn1—O1^i^	97.52 (10)	C5—C4—C3	121.3 (4)
N1—Zn1—O1^i^	91.10 (9)	C5—C4—H4	119.4
O1—Zn1—O1^i^	165.78 (15)	C3—C4—H4	119.4
N1^i^—Zn1—O2	147.44 (10)	C4—C5—C7	117.8 (3)
N1—Zn1—O2	90.82 (10)	C4—C5—C6	121.3 (4)
O1—Zn1—O2	58.40 (10)	C7—C5—C6	120.8 (4)
O1^i^—Zn1—O2	110.41 (10)	C5—C6—H6A	109.5
N1^i^—Zn1—O2^i^	90.82 (10)	C5—C6—H6B	109.5
N1—Zn1—O2^i^	147.44 (10)	H6A—C6—H6B	109.5
O1—Zn1—O2^i^	110.41 (10)	C5—C6—H6C	109.5
O1^i^—Zn1—O2^i^	58.40 (10)	H6A—C6—H6C	109.5
O2—Zn1—O2^i^	89.92 (14)	H6B—C6—H6C	109.5
N1^i^—Zn1—C1^i^	95.29 (10)	C8—C7—C5	121.6 (3)
N1—Zn1—C1^i^	119.32 (10)	C8—C7—H7	119.2
O1—Zn1—C1^i^	139.05 (12)	C5—C7—H7	119.2
O1^i^—Zn1—C1^i^	29.05 (10)	C7—C8—C2	119.7 (3)
O2—Zn1—C1^i^	101.07 (10)	C7—C8—H8	120.2
O2^i^—Zn1—C1^i^	29.36 (10)	C2—C8—H8	120.2
N1^i^—Zn1—C1	119.31 (10)	N1—C9—C10	123.3 (3)
N1—Zn1—C1	95.29 (10)	N1—C9—H9	118.4
O1—Zn1—C1	29.05 (10)	C10—C9—H9	118.4
O1^i^—Zn1—C1	139.05 (12)	C9—C10—C11	120.0 (3)
O2—Zn1—C1	29.36 (10)	C9—C10—H10	120.0
O2^i^—Zn1—C1	101.07 (10)	C11—C10—H10	120.0
C1^i^—Zn1—C1	122.60 (15)	C12—C11—C10	116.2 (3)
C1—O1—Zn1	93.6 (2)	C12—C11—C11^ii^	122.0 (3)
C1—O2—Zn1	88.6 (2)	C10—C11—C11^ii^	121.8 (3)
C9—N1—C13	116.8 (2)	C13—C12—C11	120.6 (3)
C9—N1—Zn1	122.06 (19)	C13—C12—H12	119.7
C13—N1—Zn1	120.93 (19)	C11—C12—H12	119.7
O1—C1—O2	119.4 (3)	N1—C13—C12	123.2 (3)
O1—C1—C2	119.8 (3)	N1—C13—H13	118.4
O2—C1—C2	120.8 (3)	C12—C13—H13	118.4
O1—C1—Zn1	57.36 (18)	H3A—O3—H3B	108.4
O2—C1—Zn1	62.03 (18)	H3A—O3—H3C	110.1
C2—C1—Zn1	176.3 (2)	H3B—O3—H3C	110.1
C8—C2—C3	118.8 (3)	H4A—O4—H4B	108.7
C8—C2—C1	120.7 (3)	H4A—O4—H4C	110.5
C3—C2—C1	120.4 (3)	H4B—O4—H4C	110.5

Symmetry codes: (i) −*x*, *y*, −*z*+1/2; (ii) −*x*+1/2, −*y*+1/2, −*z*.

### Hydrogen-bond geometry (Å, °)

**Table:**

*D*—H···*A*	*D*—H	H···*A*	*D*···*A*	*D*—H···*A*
O4—H4B···O4^iii^	0.85	1.93	2.761 (7)	167
O3—H3A···O2^iv^	0.85	1.93	2.777 (4)	179

Symmetry codes: (iii) −*x*+1/2, −*y*+1/2, −*z*+1; (iv) *x*, −*y*, *z*+1/2.
